# Clinical characteristics, treatment methods and prognoses of patients with oral squamous cell carcinoma in Japanese population: a single institution retrospective cohort study

**DOI:** 10.1186/s12877-020-01902-3

**Published:** 2020-11-20

**Authors:** Chonji Fukumoto, Shouhei Ogisawa, Masashi Tani, Toshiki Hyodo, Ryouta Kamimura, Yuta Sawatani, Tomonori Hasegawa, Yuske Komiyama, Atsushi Fujita, Takahiro Wakui, Yasuo Haruyama, Gen Kobashi, Hitoshi Kawamata

**Affiliations:** 1 Department of Oral and Maxillofacial Surgery, Dokkyo Medical University School of Medicine, 880 Kitakobayashi, Mibu, Shimo-Tsuga, Tochigi 321-0293 Japan; 2 Section of Dentistry, Oral and Maxillofacial Surgery, Sano Kosei General Hospital, 1728 Horigome town, Sano city, Tochigi 327-8511 Japan; 3 Department of Public Health, Dokkyo Medical University School of Medicine, 880 Kitakobayashi, Mibu, Shimo-Tsuga, Tochigi 321-0293 Japan

**Keywords:** Oral cancer, Squamous cell carcinoma, Elderly patient, Surgery, Overall survival

## Abstract

**Background:**

The status of oral cancer therapy in elderly patients in Japan, where ageing is rapidly progressing, may serve as a model for other countries with similar demographics. There is controversy over what kind of treatment should be applied and how aggressively it should be applied to very elderly patients who have exceeded the average life expectancy. Given that 85 years is approximately the overall Japanese life expectancy at birth, we considered a threshold of 85 years and hypothesized that the prognosis of oral squamous cell carcinoma (SCC) patients aged ≥85 years was not inferior to that of those < 85 years. The aim of the present study was to investigate the clinical characteristics, treatment methods, and prognoses of Japanese oral SCC patients aged ≥85 years.

**Methods:**

A retrospective cohort study was performed. The data of patients with primary oral SCC (*n* = 358) from 2005 to 2018 in our institute were extracted from electronic medical records. A total of 358 patients with oral SCC were divided into two groups (≥85 years group [*n* = 26] and < 85 years group [*n* = 332]) based on the age threshold of 85 years at the first visit. Kaplan-Meier survival analyses and Cox proportional hazard models were used to analyse overall survival (OS) and hazard ratios (HRs) according to age group, treatment, and TNM classification.

**Results:**

There was no difference in the 5-year OS rate between the ≥85 years and < 85 years groups (80.8% vs. 82.2%, *P* = 0.359). This finding was the same in the operative (94.7% vs. 85.8%, *P* = 0.556) and non-operative (42.9% vs. 33.2%, *P* = 0.762) groups, indicating that age did not affect prognosis. Mortality was lower in the operative group than in the non-operative group (adjusted HR: 0.276, 95% CI: 0.156–0.489, *P* < 0.001), suggesting that surgery is a superior method. However, non-surgical treatment was selected at a higher rate in the ≥85 years group (26.9% vs. 11.1%, *P* = 0.028).

**Conclusions:**

This study suggests the prognosis of ≥85-year-old patients was not inferior to that of < 85-year-old patients. We recommend that surgery as the first choice treatment for ≥85-year-old patients with oral SCC who can tolerate surgery should be performed.

## Background

The number of new cases of oral cancer each year exceeds 350,000 worldwide, and the cumulative risk in those under the age of 75 was reported to be 0.46 in 2018 [1]. Oral cancer develops in the tongue, mandibular gingiva, maxillary gingiva, buccal mucosa, hard palate, oral floor and lips [2]. Most oral cancers are histologically diagnosed as squamous cell carcinomas (SCCs), because the surface of oral mucosa consists of squamous epithelium. However, salivary gland tumours, malignant lymphomas, malignant melanomas, and mesenchymal tumours may also develop in oral cavity [2]. Surgery is mainly performed for the treatment of oral SCCs and sometimes combined with chemotherapy (including molecular targeted treatment), immune checkpoint inhibitor therapy and radiation. These treatments have considerably improved overall survival (OS) and progression-free survival (PFS) in patients with oral SCCs. However, some patients have shown local recurrence, cervical lymph node metastasis, or distal metastasis after the initial treatment, and it is difficult to achieve complete cure in such patients.

Japan currently has the highest life expectancy (84.2 years old: male: 81.1, female: 87.1) and healthy life expectancy (74.8 years old) at birth worldwide [3, 4]. People aged ≥65 years accounted for 26.0% of the total Japanese population in 2015 [3], and this is estimated to increase to 36.4% by 2050 [3]. Thus, the number of elderly patients with oral cancer are likely to increase in Japan; this situation may serve as a model for other countries with ageing populations. In fact, the number of oral SCC patients at our institution has increased annually since it was opened in 1974, and the age distribution shows an increasing trend (Supplemental Figure 1A and B). In many reports and reviews focused on elderly patients with different types of cancers, those aged 65, 70, or 75 years old or older were regarded as elderly [5–10]. However, a detailed investigation of patients aged over 85 years old with oral cancer has not been reported.

In clinical practice, it can be difficult to select aggressive treatment methods for elderly patients who have exceeded the average life expectancy. In general, radical treatment should be applied whenever possible, even in elderly patients. Currently, people aged over 85 years have achieved the overall Japanese life expectancy at birth [4], but clinical evidence to guide treatment choices in patients with SCC aged over 85 years is insufficient. Therefore, we hypothesized that the prognosis of oral cancer patients aged 85 years and older is not inferior to that of those under 85 years. Based on this hypothesis, we performed this study to investigate the clinical characteristics, treatment methods and prognoses of patients aged ≥85 years old with oral SCC in Japan.

## Methods

### Data sources

This study was a retrospective cohort study. The review period was from April 2005 to December 2018 at the Department of Oral and Maxillofacial Surgery, Dokkyo Medical University School of Medicine. The data were obtained from electronic medical records. The design of this study was approved by the Medical Ethical Research Committee of Dokkyo Medical University Hospital (approval ID R-22-12 J).

### Patients

The data of patients with primary oral SCC (*n* = 358) were collected during the review period. Treatments and prognoses were retrospectively examined according to electronic medical records. Based on the threshold of 85 years, which is approximately the average life expectancy in Japan, the patients were divided into two groups by age at first visit. In this study, patients aged ≥85 years at the time of the first visit were defined as the “≥85 years group”, and the patients aged < 85 years were designated as the “<85 years group” for comparison.

### Exposures and potential confounders

Age, sex, primary site, disease stage, and treatment methods, which were considered exposures, and potential confounding factors were investigated, and death or survival with recurrence/metastasis were considered outcomes. The cancer stage was classified using the International Union Against Cancer (UICC) TNM Classification of Malignant Tumours, 8th edition [2].

### Statistical analysis

Descriptive analyses were performed to assess demographics and clinical factors in the 358 patients with oral SCC at baseline. A chi-squared test or Fisher’s exact test was used to compare each categorical variable between the ≥85 years and < 85 years groups. The 5-year OS and disease-free survival (DFS) rates were analysed in all patients with or without operations and in the two age groups using Kaplan-Meier survival analyses. To obtain hazard ratios (HRs) for mortality and related factors (male vs female, ≥85 years vs. < 85 years, T stage: T3 + T4a + T4b vs. Tis + T1 + T2, N stage: N1 + N2a + N2b + N3a + N3b vs. N0, and operation vs. non-operation), univariate analyses and a multivariable analysis were performed with a Cox proportional hazard model. Two-tailed *P* values of < 0.05 were considered to be significant. IBM SPSS ver. 24.0 (IBM SPSS, Inc., Tokyo, Japan) was used for the statistical analyses.

## Results

### Characteristics and treatment methods of patients with oral SCC

The characteristics and treatment methods of the patients with oral SCC in our institute are shown in Table 1. There were 358 patients included in 207 males (57.8%) and 151 females (42.3%), and their average of age (SD) was 66.1 (14.3) years old. Of the 358 patients, 26 patients were aged ≥85 years at the time of the first visit and were defined as the ≥85 years group, and 332 patients were aged < 85 years and were designated as the < 85 years group for comparisons in further analysis. The primary site, T stage, N stage, M stage and clinical TNM stage for all patients are shown in Table 1. Most of the patients were treated by operative methods (87.7%) with or without chemotherapy and/or radiation. Some patients were treated by non-operative methods (12.3%), probably because of the patients’ intentions or local and systemic limitations for surgery.

**Table 1:** Characteristics of patients with oral SCC (*n* = 358)

**Sex, male, n (%)**	207 (57.8)
**Age, mean (SD) y**	66.1 (14.3)
**Age group, n (%)**	
< 85 y	332 (92.7)
≥ 85 y	26 (7.3)
**Primary site, n(%)**	
Tongue	177 (49.4)
Lower gingiva	71 (19.8)
Upper gingiva	47 (13.1)
Buccal mucosa	31 (8.7)
Oral floor	22 (6.1)
Lip	4 (1.1)
Palate	6 (1.7)
**T stage, n(%)**	
Tis	15 (4.2)
T1	49 (13.7)
T2	88 (24.6)
T3	75 (20.9)
T4a	123 (34.4)
T4b	8 (2.2)
**N stage, n(%)**	
N0	257 (71.8)
N1	31 (8.7)
N2b	33 (9.2)
N2c	11 (3.1)
N3b	26 (7.3)
**M stage, n(%)**	
M0	357 (99.7)
M1	1 (0.3)
**Stage, n(%)**	
Stage 0	21 (5.9)
Stage 1	46 (12.1)
Stage 2	74 (20.7)
Stage 3	69 (19.3)
Stage 4a	116 (32.4)
Stage 4b	31 (8.7)
Stage 4c	6 (0.3)
**Treatment, n(%)**	
**Operative treatment**	314 (87.7)
Surgery only	225 (71.7)
Surgery and postoperative chemotherapy	52 (16.6)
Surgery and postoperative radiation	6 (1.9)
Surgery and postoperative chemoradiation	31 (9.9)
**Non-operative treatment**	44 (12.3)
Chemotherapy	3 (6.8)
Radiation	13 (29.5)
Chemoradiation	28 (63.6)

### Comparisons of the characteristics, treatment methods, and prognoses of patients with oral SCC by age (≥85 years or < 85 years)

Table 2 shows that the percentage of females was significantly higher than that of males in the ≥85 years group (*P* = 0.001). The tongue as the primary site occurred significantly less frequently in the ≥85 years group than in the < 85 years group (6/26, 23.1% vs. 171/332, 51.5%, *P* = 0.005). Regarding TNM classification, in the ≥85 years group, significantly more patients had stage T3 or more advanced disease than stage T2 or earlier stage disease (*P* = 0.038). Regarding N classification, the rate of cervical lymph node metastasis was significantly higher in the ≥85 years group than in the < 85 years group (*P* = 0.050). Thus, many ≥85 years patients had stage III or higher, but the difference was not significant (*P* = 0.077).

**Table 2:** Comparison of the characteristics, treatment methods, and prognoses of patients with oral SCC by age

	Age < 85 years group (***n*** = 332)	Age ≥ 85 years group (***n*** = 26)	*P* value^a^
**Sex, n(%)**			
Female	132 (39.8)	19 (73.1)	**0.001**
Male	200 (60.2)	7 (26.9)	
**Primary site, n(%)**			
Tongue	171 (51.5)	6 (23.1)	**0.005**
Other^c^	161 (48.5)	20 (76.9)	
**T stage, n(%)**			
Tis + T1 + T2	146 (44.0)	6 (23.1)	**0.038**
T3 + T4a + T4b	186 (56.0)	20 (76.9)	
**N stage, n(%)**			
N0	234 (70.5)	23 (88.5)	**0.050**
N1 + N2a + N2b + N3a + N3b	98 (29.5)	3 (11.5)	
**M stage, n(%)**			
M0	332 (100.0)	25 (96.2)	0.073^b^
M1	0 (0.0)	1 (3.8)	
**Stage, n(%)**			
Stage1 + 2	135 (40.7)	6 (23.1)	0.077
Stage3 + 4a + 4b + 4c	197 (59.3)	20 (76.9)	
**Treatment, n(%)**			
Non-operative treatment	37 (11.1)	7 (26.9)	**0.028**^**b**^
Operative treatment	295 (88.9)	19 (73.1)	
**Recurrence or metastasis**^d^			
No	228 (77.3)	17 (89.5)	0.170^b^
Yes	67 (22.7)	2 (10.5)	
**Death in 5 years period**			
No	273 (82.2)	21 (80.8)	0.511^b^
Yes	59 (17.8)	5 (19.2)	

^a^ Chi-squared test

^b^ Fisher’s exact test

^c^ Other primary sites included the lower gingiva, upper gingiva, buccal mucosa, oral floor, lip, and palate

^d^ Recurrence or metastasis unknown (*n* = 44) and excluded

Non-surgical treatments (Table 2) were performed in 7 (26.9%) patients in the ≥85 years group and in 37 (11.1%) patients in the < 85 years group, respectively. These data show that surgery was significantly less frequently performed in the ≥85 years group (*P* = 0.028).

**Fig. 1:**
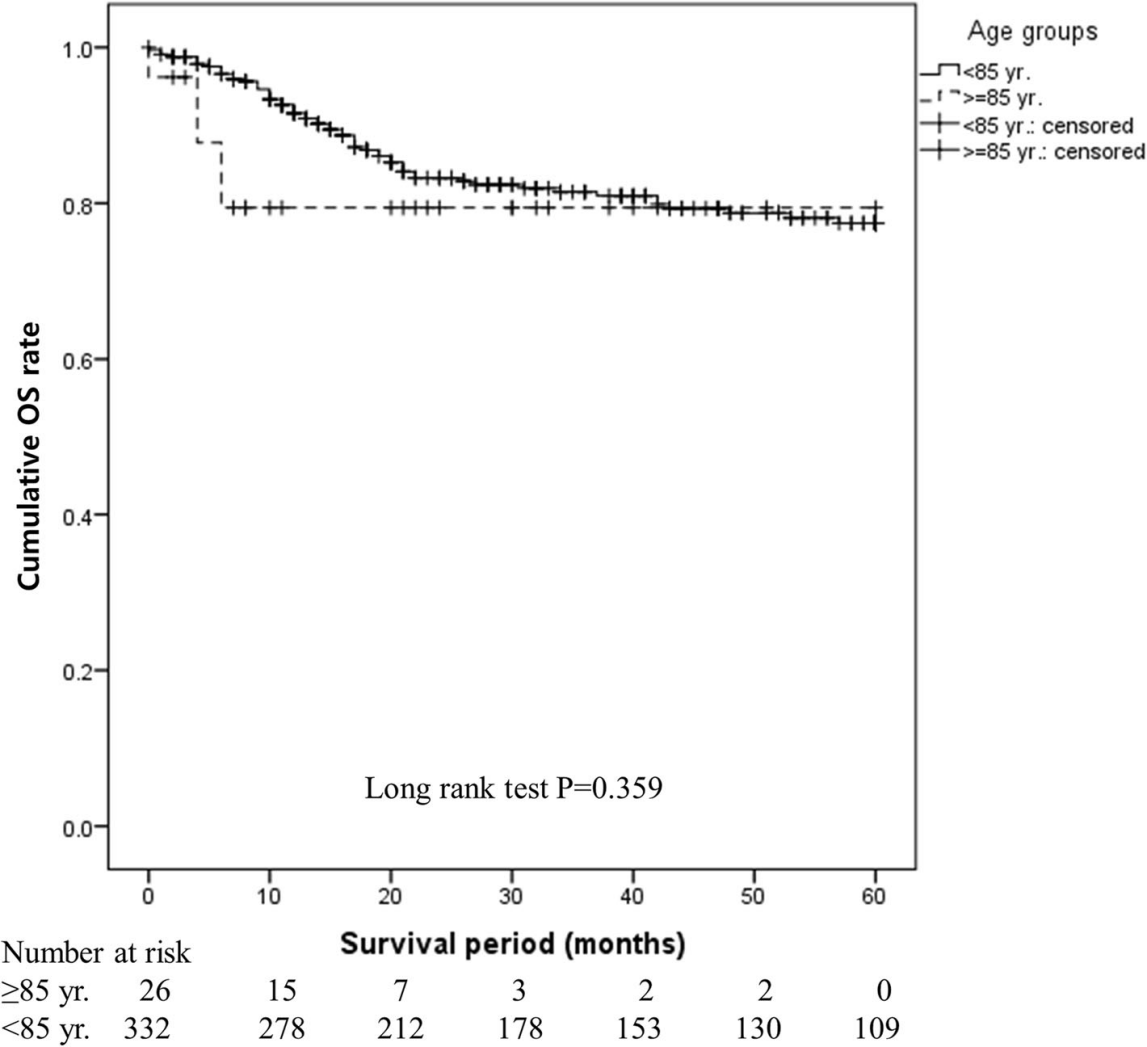
Cumulative OS rate in patients with oral SCC in the ≥85 years and < 85 years groups. OS was shorter immediately after treatment initiation in the ≥85 years group, but the 5-year OS did not differ significantly between the ≥85 years and < 85 years groups (80.8% vs. 82.2%, *P* = 0.359)

**Fig. 2:**
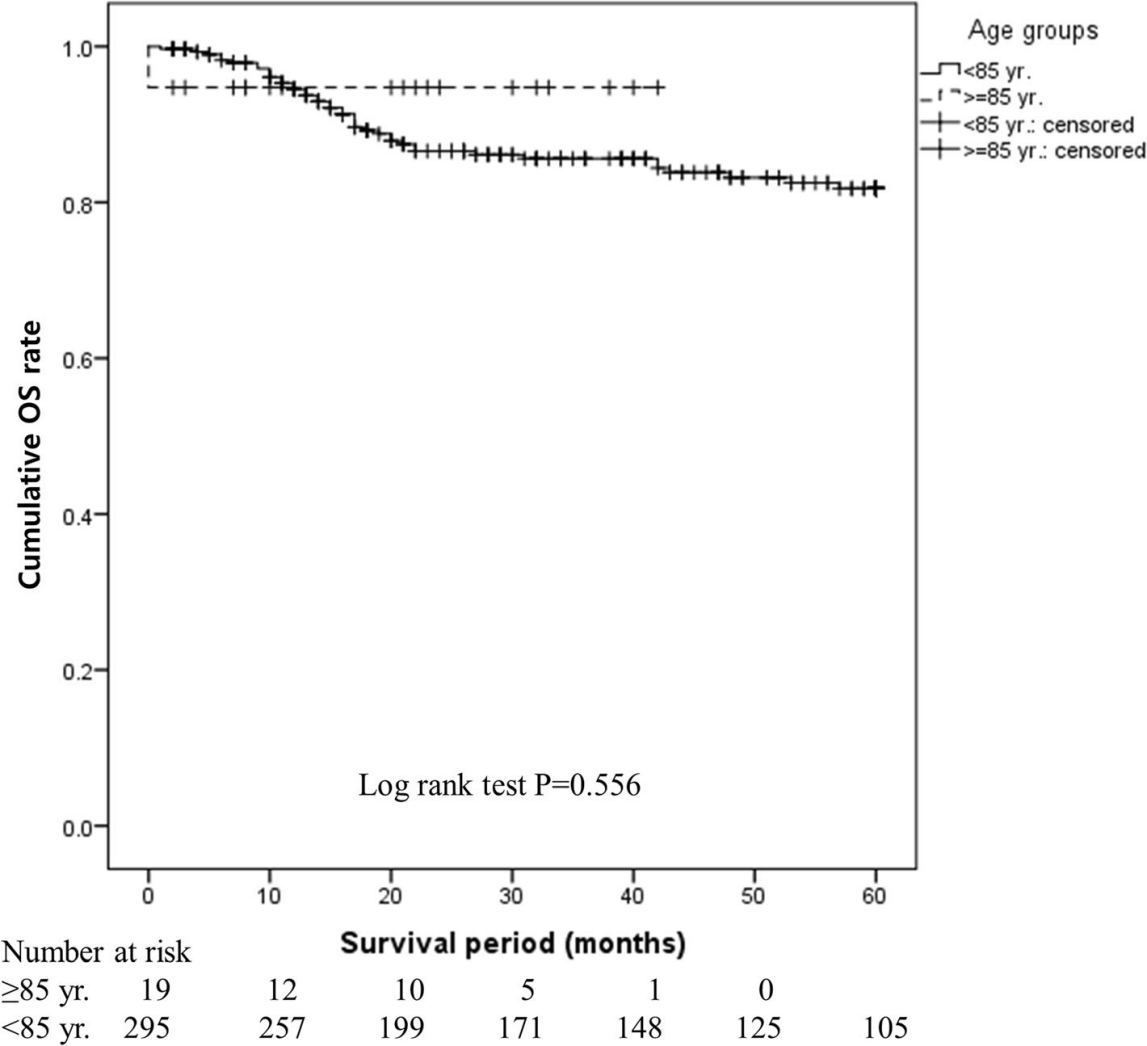
Cumulative OS rate in patients with oral SCC treated with surgery in the ≥85 years and < 85 years groups. Figure 2 shows that OS in surgical cases did not differ significantly between the ≥85 years and < 85 years groups (94.7% vs. 85.8%, *P* = 0.556)

**Fig. 3:**
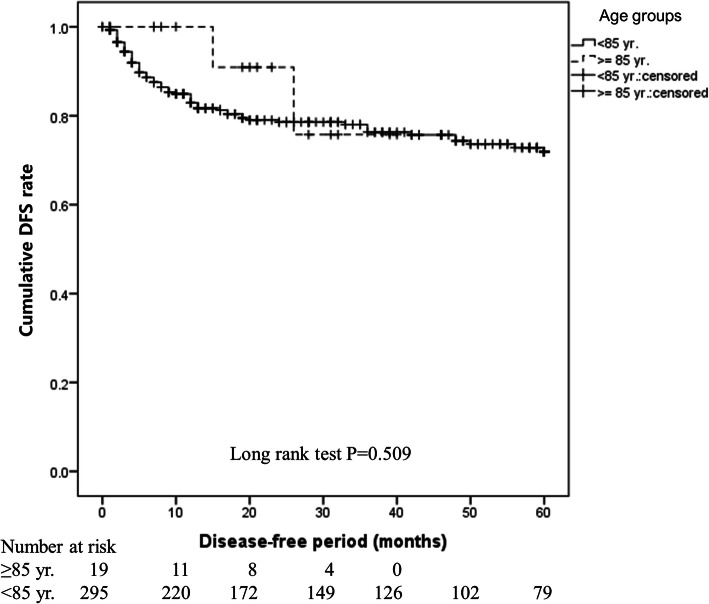
Cumulative DFS rates in patients with oral SCC treated with surgery in the ≥85 years and < 85 years groups. Figure 3 shows that DFS in surgical cases did not differ significantly between the ≥85 years and < 85 years groups (89.5% vs. 77.3%, *P* = 0.509)

**Fig. 4:**
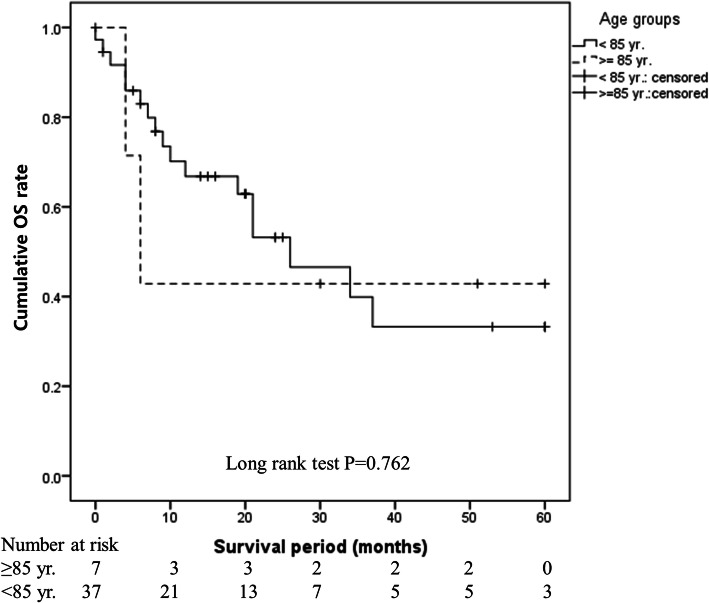
Cumulative OS rates in patients with oral SCC treated without surgery in the ≥85 years and < 85 years groups. The OS in non-surgical cases did not differ significantly between the ≥85 years and < 85 years groups (42.9% vs. 33.2%, *P* = 0.762)

OS immediately after treatment initiation was shorter in the ≥85 years group, but the 5-year OS did not differ significantly between the ≥85 years and < 85 years groups (80.8% vs. 82.2%, *P* = 0.359; Fig. 1). OS (94.7% vs. 85.8%, *P* = 0.556; Fig. 2) and DFS (89.5% vs. 77.3%, *P* = 0.509; Fig. 3) in the surgery subgroup and OS in the non-surgery subgroup (42.9% vs. 33.2%, *P* = 0.762; Fig. 4) did not differ significantly between the ≥85 years and < 85 years groups. The maximum follow-up period was 42 months in the ≥85 years group.

### Associations of mortality due to oral SCC with several factors

The mortality (Table 3) did not differ significantly in the ≥85 years and < 85 years groups in the univariate analysis (HR: 1.125, 95% CI: 0.681–1.86, *P* = 0.645) or multivariate analysis (HR: 0.900, 95% CI: 0.529–1.529, *P* = 0.696). Similarly, there was no difference in mortality based on sex or primary site. However, mortality was significantly higher in the T3 or more advanced disease group than in the T2 or earlier stage disease group in the univariate analysis (HR: 3.511, 95% CI: 1.909–6.458, *P* < 0.001) but not in the multivariate analysis (HR: 1.410, 95% CI: 0.439–4.525, *P* = 0.564). The risk of death was significantly increased in patients with lymph node metastasis in the univariate analysis (HR: 4.161, 95% CI: 2.531–6.842, *P* < 0.001) and multivariate analysis (HR: 2.660, 95% CI: 1.417–4.991, *P* = 0.002) and in surgery patients in the univariate analysis (HR: 0.183, 95% CI: 0.108–0.31, *P* < 0. 001) and multivariate analysis (HR: 0.276, 95% CI: 0.156–0.489, *P* < 0. 001).

**Table 3:** Mortality in patients with oral SCC between the non-operative and operative treatment groups during the 5-year follow-up period (*n* = 358)

	Univariate analysis					Multivariable analysis				
	Crude HR	95% CI			*P* value^a^	Adjusted HR	95% CI			*P* value^a^
**Sex, male vs. female**	1.125	0.681	–	1.860	0.645	0.900	0.529	–	1.529	0.696
**Age group, ≥85 years vs. <85 years**	1.529	0.612	–	3.821	0.364	1.172	0.395	–	3.482	0.775
**Primary site, tongue vs. other**^b^	1.015	0.622	–	1.658	0.951	1.478	0.888	–	2.460	0.133
**T stage, T3 + T4a + T4b vs. Tis + T1 + T2**	3.511	1.909	–	6.458	**< 0.001**	1.410	0.439	–	4.525	0.564
**N stage, N1 + N2a + N2b + N3a + N3b vs. N0**	4.161	2.531	–	6.842	**< 0.001**	2.660	1.417	–	4.991	**0.002**
**Stage, stage3 + 4a + 4b + 4c vs stage0 + 1 + 2**	4.201	2.139	–	8.252	**< 0.001**	1.541	0.388	–	6.118	0.539
**Treatment, operation vs non-operation**	0.183	0.108	–	0.310	**< 0.001**	0.276	0.156	–	0.489	**< 0.001**

*SCC* squamous cell carcinoma, *HR* hazard ratio, *CI* confidence interval

^a^ A Cox proportional hazard model was constructed using the variables with a *P* value < 0.1 in Table 2. M stage was not included because the percentage of M1 in those aged <85 years was zero

^b^ Other primary sites included the lower gingiva, upper gingiva, buccal mucosa, oral floor, lip, and palate

## Discussion

In this study, there was no difference in the survival rate between the ≥85 years and < 85 years groups (Fig. 1) or between the operative and non-operative groups (Figs. 2 and 3), showing that age did not affect prognosis at our hospital. The OS rate was significantly higher in the operative group than in the non-operative group regardless of age (*P* < 0.001), clearly showing that operative treatment is superior to non-operative treatment. These findings suggest that radical treatment should be applied in ≥85-year-old patients when possible and that surgery is desirable if ≥85-year-old patients can tolerate the procedure. To the best of our knowledge, this is the first report to analyse oral SCC patients aged ≥85 years, and it will be an especially useful clinical reference in the future.

We fully understand that some limitations of our study need to be considered. First, the ≥85 years group contained only 26 patients. We are aware that the sample size of this study is too small for persuasive statistical analysis. A longer study period to collect the data of a sufficient number of patients aged ≥85 years is needed. However, if we extend the review period to collect a sufficient number of patients, the therapeutic strategies and methods might change, and these changes might affect the outcome of the patients. Second, this study was a retrospective study in a single institution. Some might argue that a multi-institutional study should be conducted. However, the OS of patients with oral cancer at all stages from several institutes seems to range from approximately 55 to 65% [11, 12], while the OS at all stages in our institute was 82.1% (Table 2). Moreover, most institutions tend to avoid radical surgical treatment in patients aged ≥85 years. Therefore, a multi-institutional study of ≥85-year-old patients with oral cancer would be difficult to conduct. A strength of our study is that these results are from only a single institution that employed a consistent therapeutic strategy and had high medical standards during the study period because a single doctor (a chairman in our department at present) directed the treatment plans during this period. Additionally, variations in treatment levels and techniques, which are often observed in multi-institutional studies, did not occur.

The median age (66.1 years old) of patients with oral SCC in this study was higher than those reported in other countries [8, 9]. The proportion of females (73.1%) was significantly higher in the ≥85 years group than in the < 85 years group (Table 2). Similarly, the rate of females with oral SCC has increased with increased age in reports worldwide [7–9]. Our findings may be due to the higher life expectancy in females than in males in Japan (87.1 vs. 81.1 years) [4]) and the use of the threshold of 85 years in both sexes.

The rate of tongue cancer was significantly higher in patients aged < 85 years (Table 2), with a rate of 51.5%, than in those aged ≥85 years, whereas mandibular gingival cancer occurred most frequently in patients aged ≥85 years, with a rate of 35.7%. Pollom et al. reported similar findings that the frequent primary sites were tongue cancer and gingival cancer in younger and elderly patients, respectively [7]. In our study, the UICC TNM classification of local advancement was significantly more frequent in patients aged ≥85 years than in patients aged < 85 years, whereas cervical lymph node metastasis was seen in a low number of these patients (Table 2). A similar tendency was shown in other reports [7, 8]. Awareness of a local tumour and visiting a hospital are likely to be delayed in elderly people, and this is considered to be a cause of local advancement, but there is no clear reason for the few number of cases with cervical lymph node metastasis. There was no significant difference in the final TNM classification due to age because of the conflicting results of T/N classifications (Table 2).

In our study, the OS rate decreased immediately after treatment initiation in the ≥85 years group (Fig. 3). Of these patients, an 87-year-old female with T3N0M0 (stage III) tongue cancer suddenly died of acute heart failure 2 weeks after partial glossectomy, with no findings suggesting cervical lymph node metastasis. Vascularized free skin flap reconstruction was not performed to minimize surgical stress, and the wound was treated with primary closure. The operative time was 1 h 36 min, and blood loss was minimal. Oral ingestion was started on the day following surgery, and the course of wound healing was favourable, but cardiopulmonary arrest occurred 7 days after surgery, and the patient died despite attempted resuscitation. A risk assessment of cardiac function was performed by a cardiologist before surgery, and a radiologist suggested that there was no cardiovascular contraindication for surgery. This case illustrates the difficulty of perioperative management in ≥85-year-old patients.

Although the definition of elderly is unclear, ≥65 years old is regarded as elderly in many countries [13]. This is based on a declaration by the World Health Organization (WHO) in 1965 that when people aged ≥65 years exceed 7% of the population, society is regarded as an ageing society. The National Institute on Aging of the National Institutes of Health (NIH) classifies elderly patients into 3 groups: young old (65–74 years), older old (75–85 years), and oldest old (> 85 years) [14]. In some studies, half of new cancer patients not limited to oral cancer patients are ≥65 years old [15, 16]. The number of patients with oral SCC by year from the opening of our institute was calculated (Supplemental Fig. 1A). The median number of patients per year from 1974 to 2018 was 11. After the opening our institute, there were only a few cases per year, but this stabilized at approximately 25 patients after 2005. The number of ≥85-year-old patients per year has remained nearly constant since 2000. The numbers of patients stratified by age until 2004 and in 2005 and thereafter were calculated (Supplemental Fig. 1B). After 2005, the number of patients in their 60s increased. The peak age up to 2004 was 70–74 years old but shifted to 75–79 years old in and after 2005. Thus, the age of elderly patients with oral cancer in our institute is clearly increasing.

The selection of treatment for elderly patients requires the consideration of medical history, physical reserve capacity, physical and mental disabilities, and social background. Heterogeneity in conditions among individuals of the same age is a concern. The International Society of Geriatric Oncology (SIOG) and American Society of Clinical Oncology (ASCO) recommend the use of the Comprehensive Geriatric Assessment (CGA) in elderly cancer patients [13, 17, 18]. Many comparisons of oral cancer surgery in elderly and young patients have been reported. In an older large-scale retrospective study, the incidence of complications of surgery for cancer of the head and neck and the perioperative mortality rate were higher in elderly patients than in young patients, but the differences were small, and it was concluded that ageing should not restrict the application of surgery [19]. In a systematic review of free flap reconstruction in elderly patients with head and neck cancer, there was no difference in free flap engraftment, surgical complications, or mortality compared to these factors in young patients [20]. Non-operative treatments, such as radiation therapy and chemotherapy, are also reported to be uninfluenced by age [5, 6, 8]. Several recent cohort analyses have suggested that the side effects and treatment effects of chemoradiation therapy (CRT) are tolerable and excellent, respectively, in elderly patients with head and neck cancer [6, 8]. Huang et al. found no differences in the rates of treatment discontinuation and completion or treatment-related deaths between elderly and young patients receiving CRT [8]. In contrast, some reports illustrated difficulty in establishing indications for and the selection of treatment in elderly patients [10].

The treatment method might ultimately be decided by the patient’s intention, but we should propose the optimum treatment plan based on the clinicopathological features of the tumour, the activities of daily living (ADL) and performance status (PS) of patients, patients’ medical histories, support from patients’ families, and patients’ religious backgrounds. Although surgical resection of the tumour as the first choice treatment seems desirable, in cases in which surgery is likely to be tolerated, combinations of several novel molecular targeted therapies with surgery might further improve not only OS but also activities of daily living or quality of life after treatment in elderly patients with oral SCC. As a future task, it might be necessary not only to conduct the clinical study by increasing the number of patients aged ≥85 years but also to establish a new protocol which could objectively evaluate the conditions and the backgrounds of elderly patients with oral cancer for choosing an optimal treatment method.

## Conclusions

This study suggests that the prognosis of ≥85-year-old patients with oral SCC was not inferior to that of < 85-year-old patients. Patients aged ≥85 years had a significantly lower rate of surgical therapy than those < 85 years. The treatment of ≥85-year-old patients requires careful consideration of the condition of the patient and the results of a risk assessment. It is necessary to further compile cases to perform a more detailed analysis, but we recommend the use of operative treatment even in ≥85 years patients with oral SCC. It is desirable to use operative treatment as the first choice if psychosomatic and social conditions permit.

## Supplementary Information

**Additional file 1:** **Supplemental Figure 1A**. Number of patients with oral SCC by year in our institute. **Supplemental Figure 1B**. Number of patients with oral SCC by age. The number of patients with oral SCC by year is shown in Supplemental Figure 1A. The median number of patients per year from 1974 to 2018 was 11 (minimum: 1, maximum: 41). After opening our institute, there were only a few cases per year, but this number stabilized at approximately 25 patients after 2005 (median: 25, minimum: 12, maximum: 41). The number of patients aged ≥85 years per year has remained nearly constant since 2000. The numbers of patients by age until 2004 and in 2005 and thereafter are shown in Supplemental Fig. 1B. After 2005, the number of patients in their 60s increased. The peak age was 70–74 years old (median: 66, minimum: 27, maximum: 97) up to 2004 but shifted to 75–79 years old (median: 68, minimum: 16, maximum: 97) in and after 2005.

## Data Availability

The datasets generated and analysed during the current study are not publicly available due ethical restrictions and patient confidentiality but are available from the corresponding author on reasonable request.

## Abbreviations

SCC: Squamous cell carcinoma
OS: Overall survival
DFS: Disease-free survival
CRT: Chemoradiation therapy
