# Supplementary figures and images for: Clinical characteristics, treatment methods and prognoses of patients with oral squamous cell carcinoma in Japanese population: a single institution retrospective cohort study
Source: BMC Geriatr. 2020 Nov 20;20:487. doi: 10.1186/s12877-020-01902-3 (PMC7678127; doi:10.1186/s12877-020-01902-3)

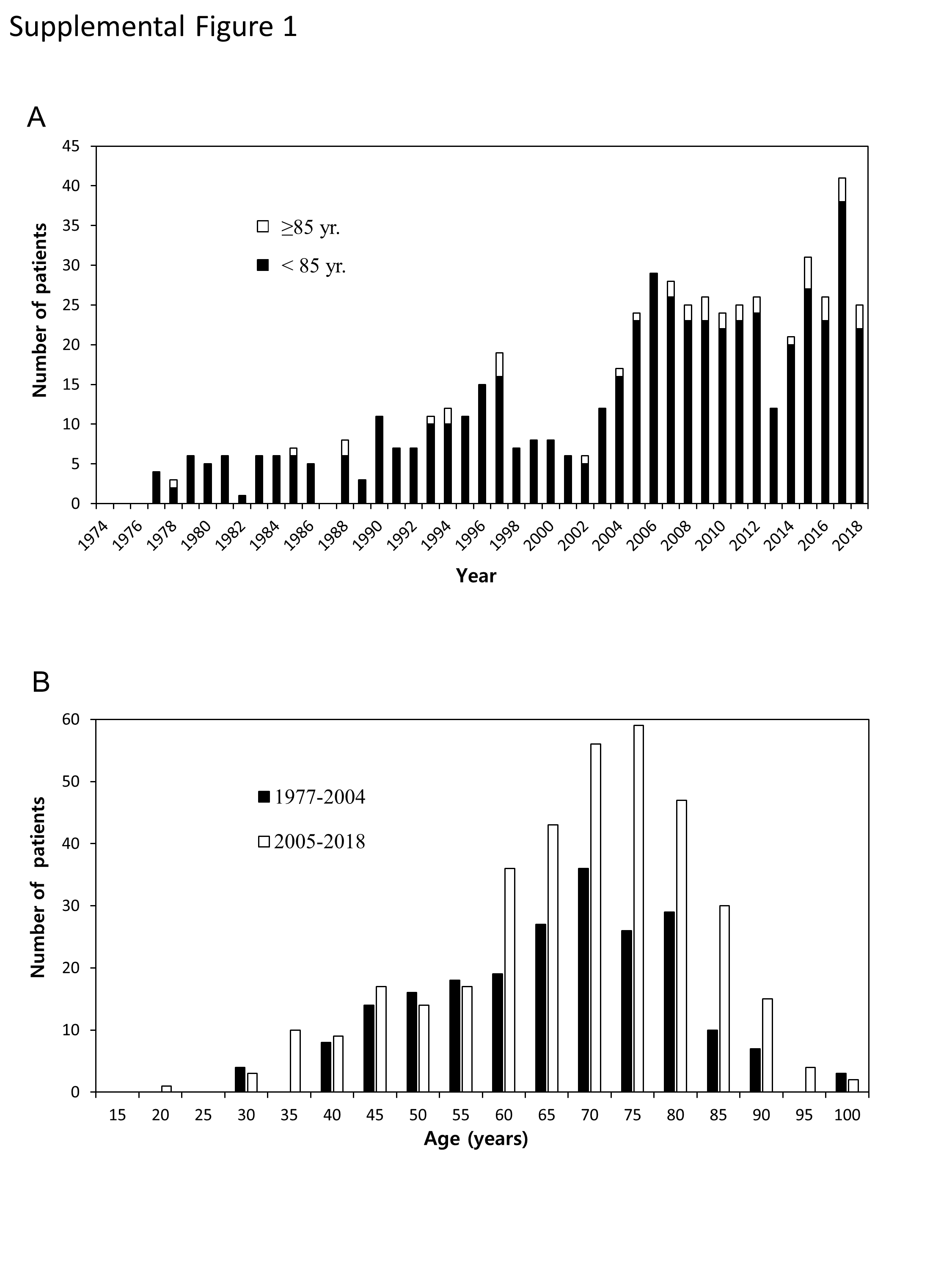

Supplement: Supplementary file 1 — Additional file 1: Supplemental Figure 1A. Number of patients with oral SCC by year in our institute. Supplemental Figure 1B. Number of patients with oral SCC by age. The number of patients with oral SCC by year is shown in Supplemental Figure 1A. The median number of patients per year from 1974 to 2018 was 11 (minimum: 1, maximum: 41). After opening our institute, there were only a few cases per year, but this number stabilized at approximately 25 patients after 2005 (median: 25, minimum: 12, maximum: 41). The number of patients aged ≥85 years per year has remained nearly constant since 2000. The numbers of patients by age until 2004 and in 2005 and thereafter are shown in Supplemental Fig. 1B. After 2005, the number of patients in their 60s increased. The peak age was 70–74 years old (median: 66, minimum: 27, maximum: 97) up to 2004 but shifted to 75–79 years old (median: 68, minimum: 16, maximum: 97) in and after 2005. [file 12877_2020_1902_MOESM1_ESM.tif]
